# Mesenchymal Stem Cells: An Overview of Their Potential in Cell-Based Therapy for Diabetic Nephropathy

**DOI:** 10.1155/2021/6620811

**Published:** 2021-03-16

**Authors:** Yan Wu, Chunlei Zhang, Ran Guo, Dan Wu, Jiayi Shi, Luxin Li, Yanhui Chu, Xiaohuan Yuan, Jie Gao

**Affiliations:** ^1^Heilongjiang Key Laboratory of Antifibrosis Biotherapy, Mudanjiang Medical University, Mudanjiang, China; ^2^Department of Physiology, Mudanjiang Medical University, Mudanjiang, China; ^3^Institute of Translational Medicine, Shanghai University, Shanghai, China

## Abstract

Diabetic nephropathy (DN) is a devastating complication associated with diabetes mellitus, and it is the leading cause of end-stage renal diseases (ESRD). Over the last few decades, numerous studies have reported the beneficial effects of stem cell administration, specifically mesenchymal stem or stromal cells (MSCs), on tissue repair and regeneration. MSC therapy has been considered a promising strategy for ameliorating the progression of DN largely based on results obtained from several preclinical studies and recent Phase I/II clinical trials. This paper will review the recent literature on MSC treatment in DN. In addition, the roles and potential mechanisms involved in MSC treatment of DN will be summarized, which may present much needed new drug targets for this disease. Moreover, the potential benefits and related risks associated with the therapeutic action of MSCs are elucidated and may help in achieving a better understanding of MSCs.

## 1. Introduction

Diabetes mellitus (DM) is a global epidemic disease affecting millions of people. According to the International Diabetes Federation (IDF), the morbidity rate of DM among adults aged between 20 and 79 years was estimated to be 9.3% in 2019, and the proportion is expected to rise to 10.9% by 2045. Moreover, the number of people with diabetes (20-79 years) will rise from 463 million in 2019 to 700 million by 2045 [1]. DM characterized with hyperglycemia may lead to the dysfunction of several major organs. Among them, diabetic nephropathy (DN) is one of the most significant microvascular complications for both type 1 and type 2 diabetic population. Previous studies have estimated that approximately 25% to 40% of those individuals living with both types of diabetes develop DN [2, 3], even when glucose control is nearly optimal, and it is the leading cause of end-stage renal disease (ESRD) [4]. In the absence of DN, the mortality rate among diabetic patients is roughly in line with that of the general population [5]. Currently, there are three possibilities for the pharmacological prevention or alleviation of chronic kidney failure: control cardiovascular risk factors (often not optimal), avoid potential renal toxins (usually unfeasible), or use causal treatment for the disease whenever possible (with unstable curative effect and frequent complications). Therefore, DN still poses a significant clinical burden despite the tremendous advances made in its diagnosis and treatment [6]. Therefore, there is an urgent need to develop safe and effective therapeutic strategies against the disease. Fortunately, regenerative medicine provides a potential strategy against DN.

Several studies have reported the potent effects of mesenchymal stem or stromal cells (MSCs) for treating kidney diseases. This has led many scientists to pursue treatment of DN using MSCs. A study conducted in 2006 reported that human bone marrow MSCs could increase pancreatic islets and beta cells that produce insulin, and decrease mesangial thickening and macrophage infiltration in diabetic mice. The study was the first to provide evidence on the potential of using restorative therapy as a cure for DN [7]. Since then, a growing number of research advances have made MSCs a viable option for DN. Specifically, research has shown that MSCs may be used to recapitulate several mechanisms that are sufficient for alleviating the progression of DN. This review will offer an overview of recent research into DN with an emphasis on the concrete mechanisms through which MSCs may enhance the functional regeneration of kidney tissues.

## 2. Diabetic Nephropathy

Diabetic complications involve the dysfunction of several organs including the heart, brain, kidney, blood vessels, peripheral nerves, eyes, and feet leading to serious health problems such as cardiomyopathy, nephropathy, peripheral neuropathy, retinopathy, and diabetic foot, respectively [8]. Such health problems are in turn associated with high morbidity rates and can result in a heavy social and financial burden. DN is a long-term major microvascular complication of type 1 diabetes and type 2 diabetes [9]. Microalbuminuria is the initial clinical hallmark of established DN followed by glomerular hypertrophy, moderate expansion of the mesangial matrix, and thickening of the glomerular capillary walls. Glomerulosclerosis is the primary structural characteristic of DN which is caused by progressive albuminuria, glomerular basement membrane (GBM) thickening, mesangial cell expansion, destabilization of podocyte foot processes, renal fibrosis, extracellular matrix accumulation, fluid retention, and blood pressure elevation [10, 11]. As the disease continues to advance, glomerulosclerosis eventually develops into irreversible end-stage renal disease over a period of years or even decades. However, the exact molecular mechanisms underlying DN progression have not yet been clearly elucidated. This has led to a lack of effective medications for DN treatment. Presently, the core of DN treatment depends on optimal control of the renin-angiotensin-aldosterone (RAAS) system using angiotensin-converting enzyme inhibitors (ACEI), angiotensin receptor blockers (ARB), or aldosterone blockers (spironolactone or finerenone) [12]. Combining ACE and ARB into a dual blockade approach decreases proteinuria, but the blockade strategies cannot reduce the risk of ESRD and also increase the risk of side effects [13]. In addition, effective control of hyperglycemia and hypertension can delay development of DN in the early stages. Newly developed hypoglycemic agents, such as dipeptidyl peptidase-4 inhibitors, glucagon-like peptide-1 receptor agonist, and sodium-glucose cotransporter 2 inhibitors (SGLT2), have been proven to have cardiovascular and renal safety and efficacy [14]. Combination therapy is also a novel choice with a previous study reporting that using RAAS blockade with SGLT2 inhibitors can protect the kidney and the heart of DN patients [15]. However, the high-risk of hypoglycemia and alterations in the pharmacokinetics of antihyperglycemic drugs should be taken into account [16]. Renal dialysis can also help in treating kidney failure, but it cannot retard the gradual deterioration of DN. Kidney transplant is also an effective method for treating ESRD. However, the immune systems of recipients may reject the transplanted organ even in instances where the patients are placed on immunosuppressive therapy. It is worth noting that only a few of the abovementioned pharmacological treatment options can mitigate the symptoms of DN. Regenerative medicine is a promising treatment option because it offers possible opportunities for restoring functionality to renal disease.

## 3. MSCs

Current research in the regenerative medical field has focused on MSCs which have been the subject of extensive investigations. Most researchers believe that MSCs are optimal candidates for cell-based treatment strategies [17]. The existence of MSCs was discovered in the late 1960s, where they were reported as occurring in the human body in mesodermal tissues. Over the years, the nomenclature of MSCs has been controversial. Currently, mesenchymal stem cells or mesenchymal stromal cells are the most commonly used terminologies for MSCs. However, some scholars have recently made a proposal to change the terminology to “medicinal signaling cells” [18, 19]. MSCs, which appear to be a native constituent of injured tissues, have emerged as a viable alternative to the standard pharmaceutical treatment modalities [20]. The following six aspects sum up the advantages of using MSCs as potential alternatives for disease treatment: [21–24] (1) MSCs are easily accessible from a variety of autologous or allogeneic adult tissues including bone marrow, adipose tissue, umbilical cord blood, skeletal muscle, synovium, spleen, thymus, lung, and amniotic fluid, and they can also be supplied by commercial providers; (2) the process of isolating MSCs is simple and rapid, and the cells can be quickly multiplied using *in vitro* systems; (3) MSCs can differentiate into a wide variety of mesodermal lineage cells and endodermal or ectodermal cells; (4) MSCs can selectively migrate to sites of injured tissues; (5) MSCs have low immunogenicity and have shown no adverse reactions whether by allogeneic or autologous engraftment; and (6) the use of MSCs does not pose any ethical controversy. The functional features of MSCs have been well exhibited in several studies, especially in studies with the aim of promoting tissue repair by means of cell-to-cell interactions [25–27]. It is worth noting that MSCs have a high metabolic activity, and their secretome processes involve the same mechanisms that are commonly described for other cell types [25]. Previous studies have shown that MSCs can secrete chemokines, cytokines, growth factors, and paracrine factors [26, 27]. In addition, the produced paracrine molecules consist of extracellular vesicles, such as exosomes [27]. Therefore, the secreted biomaterials rebuild a protective environment that enhances host cell recovery, thereby preserving or even rescuing the injured tissue from destruction. Several preclinical studies have revealed that MSCs may have a huge potential for treating a number of clinical diseases, including Alzheimer's disease, myocardial infarction, lung ischemia-reperfusion injury, and hepatic failure [28–31]. Furthermore, the versatility of MSCs has also made them an attractive candidate for clinical translation in all sorts of therapeutic applications. The most representative MSC product has gained approval to treat pediatric graft-versus-host disease (GVHD) in New Zealand, Canada, and Japan (Prochymal®; Osiris Therapeutics) [32, 33]. Additionally, recent years have seen several cell products associated with the clinical trials of other diseases [34]. Accordingly, these major developments suggest that MSC therapy will have a promising future.

## 4. MSC-Based Therapy for DN

### 4.1. Molecular Mechanisms of MSC-Based Therapy for DN

Experimental studies have demonstrated that MSCs can be used for relieving DN (Table 1). However, the exact mechanisms of DN have not been fully elucidated, and the molecular mechanisms for MSC-based therapy for DN is still under investigation. Regenerative applications for MSCs were initially heralded by their plastic ability since they are multipotent cells that have the ectopic capability of homing and differentiating into several cell types according to specific stimuli, including glomerular endothelial cells [7]. Although MSCs' homing processes are still largely unknown, studies have reported that they involve several molecules, such as chemokine receptors (CCR2, CCR4, CCR7, CCR10, CXCR5, CXCR6, and CXCR4) [35, 36], adhesion proteins, and the matrix metalloproteinase (MMP) family [37]. Among them, stromal cell-derived factor-1 (SDF-1) and its receptor CXCR4 are of great importance to MSCs and renal progenitor cell migration to a damaged kidney [38, 39]. An *in vitro* study reported that MSCs exhibited nonapoptotic membrane blebbing activity, similar to metastatic tumor cells, migrating through the endothelium and overcoming the basal barrier through the action of MMPs [40], especially MMP2 and MT1-MMP, which are essential for the migration of MSCs [41]. The expectation is that MSC infusion can have a long-term survival in the body, like hematopoietic stem cells, and accompany the individual throughout a lifetime. However, most of the studies have shown that only a small fraction of systemically administered cells can migrate to the injured tissue, and only a small percentage of the transplanted cells can differentiate into functional replacement tissue. In addition, the administered cells are almost undetectable in other organs within 24 hours. A previous study reported that some human cells were found in the glomeruli of human bone marrow MSC-treated NOD/SCID mice, but only a few of the cells differentiated into glomerular endothelial cells [7].

Currently, it is common knowledge that the MSCs can recognize and maintain the mechanical microenvironment they are exposed to by modifying their phenotype and secretome. When MSCs migrate to the injured tissue, they face a sophisticated microenvironment that features several chemical and physical stimuli that influences their biological behavior. In addition, MSCs strongly affect the organ microenvironment and local cellular dynamics, and further modulate the behavior of relevant cells [42]. The effect of MSCs is mainly mediated by secreting biologically active molecules for the reconstruction of the damaged tissues, such as transformed growth factor-*β* (TGF-*β*), vascular endothelial growth factor (VEGF), epithelial growth factor (EGF), hepatocyte growth factor (HGF), platelet-derived growth factor (PDGF), and interleukin-6 (IL-6) [25, 43]. Chang et al. reported that MSCs' conditioned medium (CM) obtained from hypoxic cultures promoted neurogenesis and restored the neurological function of rat models having traumatic brain injury using VEGF and HGF [44]. The biological relevance of these released growth factors was also justified in our previous study in which bone marrow-derived MSC (BM-MSC) conditioned medium (CM) decreased both the proliferation and extracellular matrix production of human keloid fibroblasts and attenuated skin fibrosis of a mice model [45]. The results obtained indicated that the paracrine effects induced by the MSCs played a rival role in the progress of skin repair. Several investigations have also shown that the paracrine action of MSCs decreased the deposition of fibronectin and collagen I, and cell proliferation in DN models [46, 47]. At the same time, it was also demonstrated that the cooperation among the PI3K/Akt, MAPK, and TGF-*β* signaling pathways could mediate the attenuation of DN symptoms. Among the excreted agents, exosomes derived from MSC-CM exerted an antiapoptotic effect and elevated the tight junction structure in tubular epithelial cells of damaged kidney tissue [46].

Exosomes, one of the extracellular vesicles (EVs), is an emerging approach of MSC-based therapies in tissue regeneration. Investigations have indicated that these trophic factors are released from MSCs in a free state or contained within exosomes that are naturally occurring in secreted membrane vesicles (30–40 to 100–120 nm diameter). These extracellular vesicles are believed to be important mediators of cell-to-cell communication not only through the transfer of receptors and proteins but also through the transfer of genetic information (mRNA and microRNAs) [48, 49]. Recent studies have explored the therapeutic use of exosome-derived MSCs in renal disease. Microvesicles obtained from MSC supernatants improved renal tissue injury by inhibiting TGF-*β*-mediated epithelial-mesenchymal transition (EMT) in renal proximal tubular epithelial cells (PTECs) [50]. As an important secreted agent, exosomes derived from MSCs were reported to prevent apoptosis and degeneration of tubular epithelial cells (TECs) by repressing the caspase-3 overexpression in diabetic rats. The therapeutic effect of these microvesicles in acute injury kidney models seems to be more beneficial when compared with that of conditioned medium [51], although some contradictory studies illustrated an opposite effect in chronic kidney disease in rats [52]. The autophagy induction by MSC-derived exosomes could also markedly improve renal function in a rat model of streptozotocin-induced diabetes mellitus, with a dramatic increase of light chain-3 and Beclin-1 and a significant reduction of mTOR and fibrotic marker expression [53]. In any event, these trials indicated a potential mechanism by which MSC-derived microvesicles ameliorate DN.

Taking a step forward, MSCs may provide a means for recapitulating several mechanisms in terms of preventing or treating DN, including immune-modulatory, antioxidant, and fibrosis-inhibiting mechanisms.

Inflammation, a common characteristic of an injured site, is capable of affecting the action of MSCs, and it has been recognized as a key pathogenic factor in the development and progression of DN where there is a contribution due to the imbalance of M1/M2 macrophages. In the context of sepsis, *in vivo* studies have been performed to show that MSCs can elicit macrophages to change into a M2 anti-inflammatory phenotype by secreting prostaglandin E2 (PGE2) and promoting IL-10 secretion in response to PGE2 [54]. MSCs reverted macrophages to adopt an anti-inflammatory phenotype and prevented renal injury in DN mice, which was attributed to the activation of transcription factor EB, and subsequent restoration of lysosomal function and the autophagy activity of macrophages [55]. Meanwhile, MSCs have been demonstrated to significantly decrease proinflammatory M1 macrophage-associated changes such as in IL-1*β*, IL-6, TNF-*α*, and IFN-*γ* [56]. Furthermore, MSCs are capable of suppressing and altering the function of mature dendritic cells (DCs) by reducing the development of CD103+ DC-associated transcription factors including basic leucine zipper transcriptional factor ATF-like 3, Batf3, DNA-binding protein inhibitor (ID-2, Id2), and FMS-like tyrosine kinase-3 (Flt3) [57]. This finding indicates that the immunomodulatory effect of MSCs is crucial for the success of tissue repair in the inflammatory environment of a DN setting.

Mitochondrial dysfunction is also a major pathogenic factor in diabetes-induced kidney injury. It was reported that MSC injection can suppress albuminuria and injury to TECs by improving mitochondrial function [58]. Konari et al. also demonstrated mitochondria transfer from MSCs that prevented apoptosis of impaired renal PTECs [59]. The difference between the two studies is that the mitochondria in the latter study originated from systemically administered MSCs. MSCs transferred their mitochondria to injured PTECs when cocultured *in vitro*, which rescued impaired renal cells [59]. Generally, the occurrence of an inflammatory disease is also accompanied by the release of reactive oxygen species (ROS) and the depletion of endogenous antioxidants, but antioxidant enzymes such as superoxide dismutase (SOD) are widely known to be very effective scavengers of ROS [60]. MSC-derived isolated mitochondria promoted the expression of mitochondrial SOD and Bcl-2, and at the same time inhibited ROS production *in vitro* [59]. The efficacy of mitochondria transfer from MSCs is probably a result of their ability to improve the expression of SOD followed by SOD acting to disproportionate the superoxide radical to oxygen and hydrogen peroxide, thereby protecting damaged cells against ROS generated during DN. The abovementioned study was the first study to show mitochondria transfer to rescue injured cells, which is a novel action of MSCs in DN.

**Figure 1:**
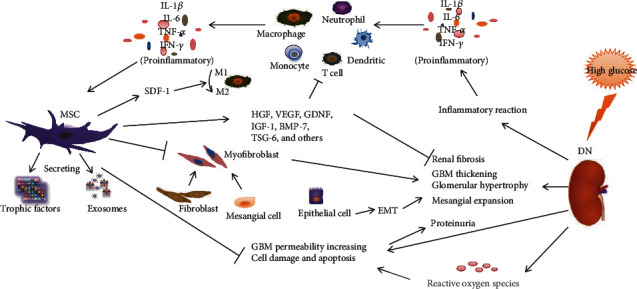
MSCs attenuate the pathological changes and clinical manifestations of DN by regulating inflammatory reaction, fibrotic response, and immune cells.

The accumulation of extracellular matrix proteins, such as the synthesis and increase of collagen type I or IV, fibronectin, and laminin, is a common feature of DN. It has been suggested that EMT contributes to the fibrotic process in DN [61]. Several studies have demonstrated that MSC delivery improves renal fibrosis in various types of kidney diseases. For example, Li et al. showed that mouse UC-MSC paracrine alleviated renal fibrosis by decreasing the deposition of fibronectin and collagen I, and elevated the levels of MMP2 and MMP9, and the mechanism may be related to TGF-*β*1-triggered myofibroblast transdifferentiation, and PI3K/Akt and MAPK signaling pathways [47]. Another study conducted on DN of a type 2 diabetes rat model reported that bone marrow-derived MSCs induced a significant inhibition of renal fibrosis, which was involved in inhibiting the TGF-beta 1/Smad3 pathway and decreasing plasminogen activator inhibitor-1 [62]. MSC administration therapy also operates through other several mechanisms, including antiapoptotic [63] and autophagy-regulating mechanisms [53]. However, it is worth noting that the mechanisms underlying these interactions are often involved at one or more levels within the complex molecular processes of DN, rather than being viewed separately (Figure 1). Arguably, cell therapy has been shown to have the most promising clinical therapeutic effects directly through tissue regeneration as well as through indirect action to enhance the natural regenerative processes on damaged and diseased tissues. Further studies on MSC action will contribute to shed light on the clinical impact of the cell-based therapy in DN. Currently, researchers are conducting preclinical and clinical studies to explore the application of MSCs to prevent the progression of DN.

### 4.2. Preclinical Studies of MSC-Based Therapy for DN

The published literature provides evidence that MSC engraftment can significantly ameliorate proteinuria serum creatinine/urea and improve renal pathological changes, including GBM thickening, glomerular sclerosis, tubule dilatation, mesangial proliferation, podocyte foot process effacement, and interstitial fibrosis. However, it is worth noting that not all the studies demonstrated a reduction in blood glucose after MSC systemic administration [64, 65], which can be probably attributed to the blood glucose control of MSC infusion delaying the progression of DN independent of direct renoprotective effects. Some studies reported that islet cell regeneration could be found in the pancreas after MSCs were engrafted. A variety of explanations, such as the animal model used, the origin of the MSC tissue, the cell dose factor, and the administration route, have been proposed to account for the phenomenon of inconsistencies in the reduction of blood glucose [66]. Moreover, these factors also have an enormous influence on other clinically relevant indicators of DN. Clarifying these factors will be critical for maximizing the efficacy of MSC therapy during application to human DN.

Rodents have always served as the primary animal model for DN experiments due to their widespread availability, definite genotypes, abundant associated experimental reagents, cost advantages, and amenability to genetic modification [67]. Almost all *in vivo* studies investigating MSCs for DN using animal models have been carried out in mice or rats. However, other animals can also be used as DN models. Pan et al. used a new DN model in tree shrews to evaluate the effect of BM-MSCs [68]. After BM-MSC transplantation, levels of glucose, triglycerides, and total cholesterol were decreased, and the levels of creatinine and urea nitrogen and 24 h proteinuria were also reduced. The study demonstrated that a tree shrew model of DN can be induced successfully with a high-sugar and high-fat diet combined with STZ injection, and BM-MSCs can alleviate the symptom of DN. Taking a step forward, An et al. developed another animal model—a rhesus macaque model of DN—and the MSCs administered in the study ameliorated the early stage of DN potentially by adjusting sodium-glucose cotransporter 2 (SGLT2) expression and resulted in improved glycemic control and anti-inflammation [69]. The two species mentioned above have greater genome homology with *Homo sapiens* when compared to rodents. Therefore, they can be used to develop an improved animal model for the study of human DN.

The different sources of MSCs may also have an effect on their action. MSCs used for research purposes are mostly obtained from bone marrow, umbilical cord, or subcutaneous adipose tissue owing to their greater accessibility. There are more studies reporting the use of BM-MSCs than those that have used umbilical cord-derived MSCs (UC-MSCs) and adipose tissue-derived MSCs (AD-MSCs). However, the clinical use of BM-MSCs faces several challenges including morbidity, pain, and low cell number during harvest. On the other hand, UC-MSCs and AD-MSCs have several advantages including higher stability in culture, higher replicative potential, lower immunogenicity, and a noninvasive harvest procedure when compared with those of other stem cells [70]. Therefore, some researchers have shifted their focus on the effect of UC-MSCs and AD-MSCs on DN. Chen et al. proved that UC-MSCs have definite therapeutic effects on DN [71]. The obtained results indicated that nephrocyte injury and albuminuria were ameliorated through their antiapoptotic property in rat models with DN when the animals received UC-MSCs. Ni et al. found that AD-MSCs could relieve renal injury in DN via activating klotho and inhibiting the Wnt/-catenin pathway [72]. In addition, Takemura et al. directly transplanted AD-MSC sheets into the kidneys of a DN rat model in order to avoid low engraftment of AD-MSCs in target organs after intravascular administration [73]. The results indicated that the method improved the engraftation efficiency and suppressed the progression of renal injury. Interestingly, another study reported that stem cells from human exfoliated deciduous teeth (SHED) significantly alleviated the pathological changes and clinical manifestations of DN in Goto-Kakizaki rats [61]. Moreover, the serum levels of inflammatory factors including IL-1 and TNF-*α* were dramatically downregulated. Additionally, the in vitro coculture of SHED with AGE-induced HK-2 cells also inhibited EMT of the epithelial cells. Therefore, SHED provides a novel potential effective therapeutic approach for attenuating DN.

MSC-based therapy has broad application prospects for the treatment of DN. However, researchers remain unsatisfied with the curative effect of the cells. For instance, the cell dose factor is a crucial aspect of cell therapy. In addition, most homing and transplantation studies only reported the observation of a small amount of MSCs upon systemic administration for long-term engraftment (>1 week) [74]. Representative studies described that the majority of transplanted MSCs (>80%) immediately accumulate in the lung tissue and then are cleared with a half-life of 24 hours [74]. Another major barrier to the effective application of MSC therapy is that insufficient MSCs are retained in injured kidneys. Therefore, Wu et al. developed SDF-1 loaded microbubbles (MBSDF-1) via covalent conjugation [75]. MSCs were intravenously transplanted after MBSDF-1 was released in the targeted kidneys in combination with diagnostic ultrasound. The obtained results indicated that the homing efficacy of MSCs to DN kidneys following the target release of SDF-1 was significantly improved at 24 hours. Zhang et al. adopted a noninvasive ultrasound-targeted microbubble destruction (UTMD) technique to enhance the homing of MSCs to kidneys, thereby improving renal repair in DN rats [76]. It is worth noting that higher doses of MSCs (4 × 10^6^ cells) may give rise to treatment-related adverse events, such as vomiting and increased respiratory rate, during transplantation of the cells [77].

In addition, some researchers have begun to attempt to promote the function of MSCs aside from considering the cell dose. Rashed et al. used new methods to increase the effect of MSCs for DN as well [78]. MSCs were pretreated with melatonin before the cells were infused into the DN model, which showed that pretreatment of MSCs with melatonin improved kidney functions compared with MSC administration alone. All the abovementioned studies provided new insights into DN therapy, which may be used as a new therapy for underlying diabetic nephropathy (DN) pathogenesis.

The MSC administration route is also a key point that should not be overlooked. The majority of studies delivered MSCs through the tail vein because the operation of the method is simple. Wang et al. demonstrated by meta-analysis that MSC therapy could induce significant effects including a greater reduction in serum creatinine and a better preserved renal function using arterial delivery than when using the intrarenal delivery and intravenous treatment [79]. This can be attributed to the fact that the MSCs delivered by intrarenal injection were just located near the site of injection, while the infused MSCs by arterial injection would enter the damaged kidney more quickly owing to the fact that the artery is rich in blood flow and blood velocity. This meta-analysis aiming at the delivery route may provide significant clues for animal experiments even for human clinical applications.

### 4.3. Clinical Research on MSC-Based Therapy for DN

MSCs are being clinically explored as a new therapeutic for treating a variety of diseases. According to the official database of the U.S. National Institutes of Health (https://clinicaltrials.gov), the majority of clinical studies currently focus on nervous system disorders and bone and cartilage diseases for MSCs registered as a potential therapy. In contrast, although a growing number of animal and in vitro studies have indicated good prospects for an MSC-based therapy in DN, only a handful of clinical studies are evaluating the regenerative and therapeutic role of MSCs in this condition now. The findings of these clinical trials serve as essential information, and the research bases for the later clinical studies are listed in Table 2 (completed and ongoing trials).

A multicenter, randomized, double-blind, dose-escalating, sequential, and placebo-controlled trial registered in ClinicalTrials.gov (NCT01843387) evaluated the safety, tolerability, and efficacy of adult allogeneic bone marrow-derived mesenchymal precursor cells (MPCs) in 30 volunteers with moderate-to-severe DN at three Australian centers. The patients received single intravenous (IV) infusion (150 × 10^6^ or 300 × 10^6^ allogeneic MPCs), and the trial duration was 60 weeks. This clinical study demonstrated that MPC infusion in patients with DN may be considered safe since there were no acute adverse events (AEs) associated with administration and no patients developed persistent donor-specific anti-HLA antibodies. In addition, a more stabilizing or improving eGFR and mGFR was observed at week 12 in the MPC-treated group, thereby providing a potential mechanistic clue to stem cell actions in this disease [80]. Most of the clinical trials are in phase I or II studies and they are aimed at assessing safety and tolerability and exploring the therapeutic effects of cell-based therapy, thus indicating that the stem cells and related therapy in clinical application are still a long way off.

## 5. The Future Directions for MSCs and MSC Alternatives in DN

MSC-based therapy is a promising alternative for the treatment of diabetic kidney disease. Despite this review presenting an overview of MSC therapy for DN, many questions have not yet been answered. The key point to consider should be how to maximize the therapeutic impact of MSCs, which may potentiate the effects by enhancing their proliferation, survival, engraftment, and paracrine properties. Several strategies have been tested to heighten the benefits, principally physical, physiological, and pharmaceutical preconditioning of stem cells, such as biological cytokine, MSC cell sheet, specific drug hypoxia, or medical equipment induction, which have created new chances that should be further explored.

In addition to promoting the therapeutic effect of MSCs indirectly, some investigations have indicated that MSCs have typically involved genetic manipulations to alleviate DN. MSCs modified with the angiotensin-converting enzyme (ACE) 2 gene could significantly inhibit renin-angiotensin system (RAS) activation and reduce glomerular fibrosis [81], yet research literature on treating DN is still limited. Notably, previous studies have reported that there is a possibility that MSCs can be modified to express some peptides or proteins with antitumor properties [82, 83]. MSCs infected with herpes simplex virus thymidine kinase (TK) gene by lentiviral transduction could exert a great antitumor effect in an animal model bearing a metastastic RIF-1 (fibrosarcoma) tumor. However, the cells did not transform their stem cell properties [82]. Moreover, nongenetic modification of MSCs by incorporating nanoparticles carrying chemotherapeutics also almost did not alter their viability, differentiation, and/or migration potential [84]. In the A549 orthotopic lung tumor model, nanoengineered MSCs loaded with the anticancer drug paclitaxel (PTX) could home in to tumors and form cellular drug depots that released the drug load over a prolonged period of time [85]. The nanoengineered MSCs exerted significant inhibition of tumor growth and better survival, despite extremely low doses of PTX, indicating that MSCs are an efficient delivery vehicle for specific drugs to enhance the efficacy of standard chemotherapy.

One of the most important action mechanisms for MSCs is mediated by exosomes. Exosomes, nanosized membranous vesicles secreted by an array of cells, were recently introduced as a new kind of drug delivery system due to their unique and important performance pharmacologically. Exosomes are closely associated with the occurrence and progression of a variety of diseases by participating in physiological processes such as cell communication, cell migration, angiogenesis, and antitumor immunity *in vivo*. And they have a high ability of penetrating organ interstitium and are endowed with a natural targeting ability due to their nanosize. MSC-derived vesicles modulate several pathways involved in the pathophysiological process of DN, including podocyte apoptosis and proliferation, inflammation response, immune regulation oxidative stress, and ECM remodeling. However, the number of exosomes released by most mammalian cells is relatively low, and their purification is very tedious, thereby leading to a relatively low yield [86]. In addition, the potential risks associated with MSC transplantation should be taken into account. However, the risks may not be observed in a short time period following administration. The long-term risks may comprise potential maldifferentiation, immunosuppression, and instigation of malignant tumor growth. Therefore, exosome-mimetic nanovesicles are very compelling for the development of a nanodrug delivery system. Nanosized cellular vesicles formed by membrane fusion after cell mechanical fragmentation feature long circulation time *in vivo*, small particle size, high tumor permeability, many kinds of encapsulated drugs (hydrophobic drugs, peptides, and nucleic acids), and slow drug release, which can achieve the effective load of nucleic acid drugs [87, 88] and also achieve the payload of chemotherapeutic drugs [89]. Currently, most experimental studies are primarily focused on short-term effects of MSC therapy, while largely ignoring the evaluation of long-term effects. This review has shown that the novel cell-free therapy based on MSCs might become an attractive alternative for the treatment of DN in future clinical applications.

It is projected that, within the next few years, the challenge for future studies will be to significantly expand our understanding of the key molecular mechanisms involved in MSC action. Elucidation of the key molecular mechanisms of MSC action will enhance their effects when applied in clinical trials; in turn, the strategy will be very conducive to reducing the morbidity and mortality of diabetic kidney disease. It is our hope that conditioned media, extracellular vesicles, and nanosized cellular vesicles will be used as cell-free substitutes for MSCs. In addition, conducting large experimental studies, registered clinical trials, and individual-data meta-analysis will help us understand and determine optimal cellular and subcellular therapies to attenuate diabetes-induced kidney injury.

## 6. Conclusions

DN remains a major clinical complication of diabetes mellitus patients, as it reduces the quality of life and overall survival. Increased academic research has focused on the effectiveness of MSCs in the treatment of DN due to the lack of clinical effective therapeutic strategies and the recent advances for MSC therapy in regeneration medicine field. However, the safety profile of MSC-based therapy needs further research, as standardized approaches for MSCs are not yet developed, along with the optimal dosage, time, and route of administration. Furthermore, the therapeutic effect of MSCs in preclinical studies has not yielded satisfying outcomes. These challenges have made it difficult to visualize the use of the MSC-based therapy in clinical implementation in the short run. However, cell-free therapy based on MSCs or therapeutic genes of modified MSCs may become a future trend of development. The safety and effectiveness MSC products have not been officially recognized despite the approval for marketing of several MSC products in some countries. However, listing does not mean that MSC research has been terminated. The use of MSCs is fascinating because it always gives us some surprises, and thus the mystery behind these surprises should be elucidated. In conclusion, MSC-based therapy has a high potential for managing DN. However, the challenges associated with the therapy must be addressed before its application can be incorporated in clinical settings. Therefore, there is still a long way to go before such cell therapy can be used in clinical practice.

## Figures and Tables

**Table 1:** Representative research on DN treated with MSC-based therapy in preclinical studies.

Study	Model	Cell type	Route	Efficacy results
Li et al., 2020 [47]	Mice	UC-MSCs	IV	Albuminuria, glomerulus injury, and fibrosis were alleviated in DN mouse models after repeated injection with mUC-MSCs
Lang and Dai, 2016 [62]	Rats	BM-MSCs	IV	BM-MSCs significantly suppressed renal fibrosis by reducing PAI-1 protein and decreasing ECM accumulation in rats with DN
Liu et al., 2020 [81]	Rats	BM-MSCs	IV	MSCs modified with ACE2 can inhibit renal RAS activation and reduce glomerular fibrosis
Zhang et al., 2020 [57]	Rats	BM-MSCs	IV	MSCs possessed the protective effect on the kidney of DN rats, which may be related to CD8(+) T cell immunosuppression mediated by CD103(+) DCs
Yuan et al., 2020 [55]	Mice	BM-MSCs	IV	MSCs suppressed inflammatory response and ameliorated renal injuries in DN mice via TFEB-dependent M1/M2 macrophage (M*φ*) switch
Chen et al., 2020 [71]	Rats	UC-MSCs	IV	UC-MSCs inhibited 24-hour urinary total protein, urinary albumin to creatinine ratio, serum creatinine, and blood urea nitrogen, attenuated pathological abnormalities and reduced the apoptosis of renal cells in DN rats
Takemura et al., 2020 [73]	Rats	AD-MSCs	Under renal capsule	AD-MSC sheet engraftment directly into the kidney improved transplantation efficiency and inhibited renal injury progression
Lee et al., 2019 [58]	Mice	UC-MSCs	IV	MSCs prevented the progression of DN by reversing mitochondrial dysfunction in TECs via the induction of arginase-1 in macrophages
Konari et al., 2019 [59]	Rats	BM-MSCs	IV	BM-MSCs transferred their mitochondria to damaged proximal tubular epithelial cells and also have shown a potentially therapeutic effect on DN
Ebrahim et al., 2018 [53]	Rats	BM-MSCs	IV	MSC-derived exosomes could exhibit the potential nephroprotective effects of a DN model by upregulating autophagy associated with suppression of mTOR pathway
Lv et al., 2014 [90]	Rats	BM-MSCs	IV	MSC injection attenuated glomerular injury in streptozotocin-induced DN model via inhibiting oxidative stress
Rashed et al., 2018 [78]	Rats	BM-MSCs	IV	BM-MSCs pretreated with melatonin enhanced its proliferation and efficiency, and ameliorated kidney functions in a rat model with DN
Wu et al., 2014 [75]	Rats	BM-MSCs	IV	Destruction of ultrasound-targeted SDF-1-loaded microbubbles could promote MSCs homing to early DN kidneys
An et al., 2019 [69]	Rhesus macaque	BM-MSCs	IV	MSCs could ameliorate a rhesus macaque model of DN by influencing SGLT2 expression, glycemic control, and anti-inflammation
Pan et al., 2014 [68]	Tree shrews	BM-MSCs	IV	MSC transplantation could home to injured kidneys and pancreas, and reduced 24 h proteinuria and improved insulin resistance of a tree shrew model with DN
Rao et al., 2019 [61]	Rats	SHED	IV	SHED attenuated DN by inhibiting advanced glycation end product-activated EMT
Ni et al., 2015 [72]	Rats	AD-MSCs	IV	AD-MSC engraftment might alleviate renal injury in DN by activating klotho and inhibiting Wnt/*β*-catenin pathway
Wang et al., 2013 [65]	Rats	BM-MSCs	Intra-arterial injection	MSCs attenuated podocyte injury and albuminuria in a type 1 DN rat model by mediating in part the increase of BMP-7 secretion

**Table 2:** Ongoing or completed clinical trials with MSC-based therapy in DN.

ClinicalTrials.gov identifier	Cell type	Subject number	Cell dosage	Route	Trial status
NCT01843387	BM-MPC	30	1 dose: 150, 300 × 10^6^ cells	IV	Completed
NCT02585622	BM-MSCs	48	1 dose: 80, 160, 240 × 10^6^ cells	IV	
NCT04216849	UC-MSCs	54	5 doses: 1.5 × 10^6^ cells/kg	IV	Phase 2
NCT03288571	UC-MSCs	20	3 doses: 1 ml cell suspension	Renal parenchyma	Phase 2
NCT03840343	AD-MSCs	30	2 doses: 2.5, 5 × 10^6^ cells/kg	Intra-arterial delivery	Phase 1
NCT04125329	UC-MSCs	15	3 doses: 1 × 10^6^ cells/kg	IV	Early phase 1

## Abbreviations

ACE: Angiotensin-converting enzyme
ACE2: Angiotensin-converting enzyme 2
ACEI: System using angiotensin-converting enzyme inhibitors
AD-MSCs: Adipose tissue-derived mesenchymal stem cells
AEs: Adverse events
ARB: Angiotensin receptor blockers
BM-MPC: Bone marrow-derived mesenchymal precursor cell
BM-MSCs: Bone marrow-derived mesenchymal stem cells
BMP-7: Bone morphogenetic protein-7
CCR: Chemokine receptors
CM: Conditioned medium
DCs: Dendritic cells
DM: Diabetes mellitus
DN: Diabetic nephropathy
ECM: Extracellular matrix
EGF: Epithelial growth factor
EMT: Epithelial-mesenchymal transition
ESRD: End-stage renal diseases
EVs: Extracellular vesicles
Flt3: FMS-like tyrosine kinase-3
GBM: Glomerular basement membrane
GDNF: Glial cell line-derived neurotrophic factor
GVHD: Graft-versus-host disease
HGF: Hepatocyte growth factor
IFN-*γ*: Interferon-*γ*
IGF-1: Insulin-like growth factor 1
IL-1*β*: Interleukin-1*β*
IL-6: Interleukin-6
IV: Intravenous
MBSDF-1: SDF-1-loaded microbubbles
MMPs: Matrix metalloproteinases
MSCs: Mesenchymal stem or stromal cells
PDGF: Platelet-derived growth factor
PGE2: Prostaglandin E2
PTEC: Proximal tubular epithelial cells
PTX: Paclitaxel
RAAS: Renin-angiotensin-aldosterone system
RAS: Renin-angiotensin system
ROS: Reactive oxygen species
SDF-1: Stroma cell-derived factor 1
SGLT2: Sodium-glucose cotransporter 2 inhibitors
SHED: Stem cells from human exfoliated deciduous teeth
SOD: Superoxide dismutase
TEC: Tubular epithelial cell
TFEB: Transcription factor EB
TGF-*β*: Transformed growth factor-*β*
TK: Thymidine kinase
TNF-*α*: Tumor necrosis factor-*α*
TSG-6: Tumor necrosis factor *α* stimulated gene 6
UC-MSCs: Umbilical cord-derived mesenchymal stem cells
UTMD: Ultrasound-targeted microbubble destruction
VEGF: Vascular endothelial growth factor.
